# Charter and Recognition of the Cincinnati College

**Published:** 1837

**Authors:** 


### APPENDIX.

### CHARTER AND RECOGNITION OF THE CINCINNATI COLLEGE.

When the Departments of Law and Medicine of the Cincinnati College were about to go into operation, in the Autumn of 1835, it was found that among the reports, intended to arrest them, was this, that the charter of the institution did not authorize their establishment. As the effect of this report on the minds of students was not known, till the classes were made up, no adequate corrective could be applied in time; but in reference to the future the following documents were obtained. They were not made public during the next vacation however, because the friends of the college could not believe, that its enemies would have the disingenuousness, to repeat the statements of the preceding year. But emboldened by the forbearance of the Board, their assertions were reiterated with increased energy, in the Autumn of 1836, and had the effect of turning from the institution, many young gentlemen from a distance, who came to Cincinnati for the purpose of receiving its instruction. In addition to this injury, another, affecting equally the character of the trustees and professors, was inflicted, in the suspicion excited abroad, that they were capable of organizing schools not authorized by their charter; and of thus practising an imposition on the community. It is under these circumstances, that the Faculty of Medicine, with the permission of the Board, have determined to give publicity to the following

### LEGAL DECLARATIONS RELATIVE TO THEIR CHARTER.

At a meeting of the Trustees of the Cincinnati College, held in the College Chapel, December 13, 1836, the following preamble and resolutions were adopted:

*Whereas* the Trustees of the Cincinnati College, have been repeatedly informed, that certain persons have circulated, among students of Law and Medicine, the assertion, that the *charter* of this institution (expressly conferring all the powers usually exercised by any other College or University in the Union) does not authorize the establishment of Law and Medical Schools or Departments; and *whereas*, such representations are calculated to injure or retard the prosperity of the College; therefore

*Resolved*, That the Professors of Law in the said Institution, be respectfully requested to furnish this Board with a written statement of their views on the following questions:

1st. Does the Charter of the Cincinnati College empower the Board of Trustees to appoint Professors of Medicine and Law; and authorize those sciences to be taught in the Institution?

2d. Does it empower them to confer the Degree of Doctor of Medicine, and that of Bachelor of Laws?

3d. Does the Charter, in any way, prohibit them from establishing schools for the regular education and graduation of physicians and attorneys at law?

A true copy: P. S. SYMMES, Sec.

The undersigned, Professors of Law in the Cincinnati College, in compliance with a resolution of the Board of Trustees, adopted December 13, 1836, submit the following opinion upon the questions therein propounded.

1st. Does the Charter of the Cincinnati College empower the Board of Trustees to appoint Professors of Medicine and Law, and authorize these sciences to be taught in the Institution?

To this question we reply in the affirmative. The Charter contains the following provisions: "The object of this act of incorporation is hereby declared to be the erection and maintenance of a College."

"The Board of Trustees may elect a President and Vice President of the College, and *may appoint such Professors and Tutors as they shall think necessary.*"

"They may, from time to time, make and enforce such rules, regulations and by-laws, for the government and well-being of the College, as may seem to them proper."

"They may appoint a Faculty to consist of the President, Vice President, Professors, and such other persons as they may judge necessary, and may vest in the Faculty so appointed, such powers as they may think expedient."

"They may grant and confer on any candidate, in such form as they may direct, *all or any of the degrees*, that are usually conferred in any College or University within the United States."

"They may cause the principles of morality and of the Christian religion to be inculcated; but the religious tenets that may be peculiar to any particular sect or denomination, shall never be taught or enforced in the College."

These are the only provisions of the charter which have an immediate bearing upon any of the questions submitted to us. They expressly authorize the Board of Trustees to "*appoint such professors and tutors as they shall think necessary.*" If, therefore, the Board think the appointment of *Professors of Law and Medicine*, necessary, their power to appoint such professors, is beyond a doubt. It is expressly conferred in the clearest possible language; and there is no prohibition or restriction. The only reference, in the Charter, to the branches of knowledge which may be taught in the College, is a prohibiiion, above quoted, of teaching *sectarian religion;* and this very restriction operates as a virtual authority to teach every other branch of knowledge, in which *Medicine* and *Law* are of course included. On the first question, then, there cannot be a shadow of doubt.

2d. Does the Charter empower them to confer the Degree of Doctor of Medicine, and that of Bachelor of Laws?

To this question, also, we reply in the affirmative. The Charter authorizes them to confer "*all or any of the degrees, that are usually conferred in any College or University within the United States.*" The only question then, is, are the degrees of *Doctor of Medicine* and *Bachelor of Laws*, usually conferred in any College or University in the United States? This is a question of fact, with which we are not concerned. We only say, that if such degrees are elsewhere conferred, the power to confer them here cannot be doubted, because it is expressly given; and we see not how more clear and comprehensive language could have been used.

3d. Does the Charter in any way prohibit them from establishing schools for the regular education and graduation of physicians and attorneys at law?

To this question we reply in the negative, as we understand its terms. By "*schools*," we suppose, are meant *departments* of the College. If so, there is not only no prohibition against creating medical and law departments, but the power to create them, is included in the provisions above quoted, as the less is always contained in the greater. These departments are created by *by-laws;* and the power to make by-laws, and carry them into execution, is conferred in the broadest terms. Besides, on general principles, the power to do a given thing, always implies the power to use the proper means. Therefore, the power to cause the branches of law and medicine to be taught in the College, implies the power to organize distinct departments for that purpose. There is no more doubt, in short, of the power to create medical and law departments, and to confer degrees in medicine and law, than there is of the power to create an academical department, and confer the ordinary academical degrees.

Cincinnati, January 18, 1837.

JOHN C. WRIGHT,

T. WALKER,

EDWD. D. MANSFIELD,

JOS. S. BENHAM.

We, the undersigned, members of the Cincinnati Bar, having examined the "Charter of the Cincinnati College," do concur in the above opinion, as expressed by the Professors in the Law Department of that institution:—

| | |
|---|---|
| E. S. Haines, | James Hall, |
| Daniel J. Caswell, | N. C. Read, |
| Edward Woodruff, | L. M. Gwynne, |
| Ben. M. Piatt, | Samuel Eells, |
| Adam N. Riddle, | U. Tracy Howe, |
| W. M. Corry, | James H. Perkins, |
| John G. Worthington, | Daniel Van Matre, |
| Crafts J. Wright, | W. R. Morris, |
| Charles Fox, | J. W. Piatt, |
| James Southgate, | Elisha W. Chester, |
| Robert T. Lytle, | Talbot Jones, |
| Edward P. Cranch, | Rufus Hodges, |
| B. T. Wright, | B. Drake, |
| William Rankin, Jr., | S. P. Chase. |

In reference to those who have given the foregoing opinions, two facts may be stated. *First*, Judge Wright, whose name stands at the head of the column, was, at the time of signing the paper, the *President* of the Board of Trustees of the *Medical College of Ohio*; the professors of which were the persons who undertook to decry our Charter; *second*, the gentlemen who have signed the paper, compose at least three-fourths of the Cincinnati Bar; and none to whom it was presented, refused to subscribe it.

### RECOGNITION BY OTHER SCHOOLS.

University of Virginia, September 11, 1835.

*Sir*,—The Faculty of the University of Virginia have had under consideration your letter in regard to the "Cincinnati College," and I am directed to say that we have, by an unanimous vote, recognised your Institution "as technically equal to the other medical institutions of the country."

Very respectfully, your obedient servant,
ALFRED T. MAGILL, M. D., Prof. Med., &c.

WM. R. MORRIS, Esq., Pres. B. of T. C. C.

---

Baltimore, August 11, 1835.

*Dear Sir*,—In reply to your communication of the 3d instant, requesting to know whether the Medical Department of the Cincinnati College will be regarded, by the Faculty of the University of Maryland, as equal, technically considered, to the other Medical Schools of the United States, I have great pleasure in informing you, that the Faculty of the University of Maryland will consider one Session passed in the Cincinnati College, as technically equal to one passed in any of the other schools of the Union.

I may perhaps be excused in expressing my regret at one of the regulations of your College, which requires that a College to be technically equal to your own, shall have not less than five professors. This rule would exclude the students of one of the most useful institutions in the Union—the University of Virginia,—which, although it has but three medical chairs, has a session of ten months' duration. Its students are admitted in both the Universities of Pennsylvania and Maryland.

I am, your obedient servant,
ROBLEY DUNGLISON,
Dean of the Med. Faculty of the University of Maryland.

WM. R. MORRIS, Esq., Pres. B. T. C. C.

---

Philadelphia, September 7, 1835.

*Dear Sir*,—In consequence of the absence of the Faculty of Jefferson Medical College from town, they have not till now been able to decide on the subject of your letter. They agree to state that the Cincinnati College will be considered by them as technically equal to the other Colleges of the United States in the transaction of the business of the College, as far as regards the proposed degree.

Yours truly, with many wishes for the success of your institution,

WM. R. MORRIS, Esq. S. COLHOUN, Dean.

Philadelphia, July 3, 1837.

*Dear Sir*,—Doctor Hayes exhibited to me a few days ago a letter from you, of June 16, making inquiry whether the Cincinnati Medical College was admitted to the *Ad Eundum* of the University of Pennsylvania? To this, I have the honor to reply in the affirmative. I am, very respectfully, your obedient servant,

Prof. D. DRAKE. W. E. HORNER, Dean, &c.

[A verbal recognition of the same kind had been made in the autumn of 1835.]

---

Medical Institution of Yale College, July 15, 1837.

I hereby certify, that at a meeting of the Faculty of the Medical Institution of Yale College, July 14, at the house of Dr. Eli Ives, Professor of the Theory and Practice of Medicine, a letter was laid before the Board by me from Dr. Daniel Drake, requesting that a resolution might be passed by said Board, recognizing a course of lectures in the Medical School of the Cincinnati College, "as technically equal to one in the Medical Institution of Yale College." Thereupon, it was considered, that as our fundamental laws require the admission desired, in all cases of medical schools legally constituted, and in which an equivalent course of instruction is given; and the Board of Examiners having always acted upon this plan by admitting to an examination and to the honors of this institution all candidates coming with proper credentials from other medical schools; therefore, we, the Medical Professors of Yale College, are unanimously of opinion that the Medical School of the Cincinnati College will be regarded in the same light by the full Board of Examiners, although half of that Board, being medical men residing in other parts of the State, are now absent, and cannot be now consulted.

(Attest) B. SILLIMAN Prof. of Chemistry &c.,

Dr. DANIEL DRAKE. Medical Institution, Yale College.

---

Boston, July 7, 1837.

*Dear Sir*,—I have the honor to acknowledge the receipt of a copy of the Catalogue of the Officers and Students of the Cincinnati College, addressed to the Dean of the Faculty of Medicine in Harvard University, accompanied with a letter to Professor Bigelow of this Faculty.

I have the pleasure to send in answer to the request contained in the letter referred to, a copy of one of the provisions of the statutes of our University:—

"He shall have attended two courses of the Lectures delivered at the Massachusetts Medical College, by each of the Professors. Except that if he have attended a course of similar Lectures in any other College or University, the same may take the place of one of the above courses."

This article sets forth to what extent other Universities and Colleges are recognised here, and under what limitations the lectures of such will be received as equivalent to those in Harvard University.

I have the honor to be, Yours, &c.,

WALTER CHANNING,

Professor in Harvard University, and Dean of the Faculty of Medicine.

DANIEL DRAKE, M. D., Prof. in Cincinnati College.

Our friends of *Transylvania* laid the application of the President of our Board, made nearly two years ago, on their table for consideration; and have not yet been able to come to a conclusion what answer to give us. Meantime, they have graduated, after one course in their University, Mr. J. A. McReynolds, of Todd county, Kentucky, the only student of ours who has attended their lectures; while five of theirs have been graduated by us.

The Faculty of the *Medical College of Ohio*, have, also, neglected to recognize us. None of our pupils, however, have applied to them; but, of theirs, sixteen have been graduated in the CINCINNATI COLLEGE!

We are compelled to publish these various facts, in self-defence, and that our brethren, in distant parts of the Union, may know something of the dangers which have beset our enterprise while yet in its cradle. If it has not, like the infant *Hercules*, strangled the invading serpents, their fangs have at least not wounded its vital organs, *et quidam ex eis procul jam serpebant*. EDITORS.

